# Examining Youth Flexible ACT Model Implementation in the Netherlands

**DOI:** 10.1007/s10597-024-01260-z

**Published:** 2024-03-22

**Authors:** Marieke Broersen, Nynke Frieswijk, Maaike van Vugt, Ad A. Vermulst, Daan H. M. Creemers, Hans Kroon

**Affiliations:** 1 GGZ Oost Brabant, Oss, The Netherlands; 2 Tranzo - Tilburg School of Social and Behavioral Sciences, Tilburg University, Tilburg, The Netherlands; 3 Accare, Groningen, The Netherlands; 4 Trimbos Institute, Utrecht, The Netherlands

**Keywords:** Model fidelity, Adolescent Mental Health, Flexible Assertive Community Treatment, Integrated Care Approach

## Abstract

Model adherence is a key indicator of mental health care quality. This study investigates the degree of model adherence, as well as content and staging of care, among the first Youth Flexible Assertive Community Treatment (ACT) teams in the Netherlands. Model fidelity was assessed in sixteen teams with the Youth Flexile ACT model fidelity scale (2014 version). Mental health workers completed a ‘content of care questionnaire’ to map the interventions applied in the teams. Model fidelity scores revealed that twelve teams adhered to the Youth Flexible ACT standard with ‘optimal implementation’ (≥ 4.1 on a 5 point scale) and four teams with ‘adequate implementation’. Most disciplines were well integrated within the teams; however, several items regarding the involvement of specific disciplines and the availability of treatment interventions (peer support worker, employment and education specialist and programs, family interventions, integrated dual disorder treatment) scored below the optimum. Frequency of contact during ACT and the use of Routine Outcome Monitoring instruments scored below the optimum as well. The ‘content of care’ data showed that most clients received an individual psychological intervention, and nearly half of the client sample received scaled-up / intensified ACT care. The findings indicate a predominantly successful translation of care from the theoretical Flexible ACT framework into practice, covering both ACT and non-ACT functions. Further room for improvement lies in the incorporation of specialized disciplines in the personal and social recovery domains, including the peer support worker and employment and education specialist, as well as in specific protocolled interventions.

**Supplementary Information:**

The online version contains supplementary material available at 10.1007/s10597-024-01260-z.

## Introduction

Due to the fragmented nature of care services, children and adolescents with enduring and interrelated psychiatric- and social care needs experience barriers to accessing and engaging in regular office-based mental healthcare services (Anderson et al., 2017; Markoulakis et al., 2023). This can lead to inefficient care processes with care discontinuity and even care avoidance (Borah et al., 2021; Reneses et al., 2022; Skehan & Davis, 2017). Multidisciplinary Youth Flexible Assertive Community Treatment (Flexible ACT) teams address the multifaceted needs of these young people (0–24 years of age) in an integrated manner. These teams provide long-term assertive outreach care across several domains, encompassing psychiatric, addiction, and supportive care. The teams adjust the intensity of care flexibly through individual case management and intensive team care, thereby fostering continuity and engagement in care (Broersen et al., 2022). The Youth Flexible ACT model is an adapted variant of adult Flexible ACT (Van Veldhuizen, 2007), which is the standard service delivery model for people with severe mental illness in the Netherlands. Youth Flexible ACT has gained popularity in the Netherlands over the past decade, growing from 15 teams in 2014 to around 80 teams in 2022 that are either active or in development.

The degree to which a team implements the Flexible ACT model, termed model fidelity, is an important metric. First, high model fidelity is associated with better adult patient outcomes (Bond & Drake, 2015; Nielsen et al., 2021; Nugter et al., 2016; Van Vugt et al., 2011). Second, model fidelity provides transparency for clients, professionals, and financiers (insurance companies and municipalities). Third, assessment of model fidelity supports quality assurance of the delivered care, as it guides teams in their initial implementation and supports teams in remaining faithful to the model. Finally, as the number of Youth Flexible ACT teams increases, the risk of program drift rises. Program drift indicates issues in implementing or sustaining the Flexible ACT model, with the model diluting, as seen in ACT teams (Thorning & Dixon, 2020; Westen et al., 2021). Periodically monitoring model adherence is therefore essential. This study provides the first overview of the implementation of the Youth Flexible ACT model, focusing on data from the Netherlands.

The integration of evidence-based interventions is a pivotal aspect of the Flexible ACT framework, prominently positioned within the hourglass model (Van Veldhuizen & Bähler, 2013). The hourglass model describes three stages of care tailored to the clients’ situation and proposes interventions that align with each stage. 1) In the stabilization stage, the team works in a problem-oriented, crisis-resolution manner and has a directing role. 2) In the treatment stage, efforts are made to reduce symptoms and improve coping through psychological interventions, medical treatment, addiction interventions, or skills training. 3) In the recovery stage, the client (and the client system) is in control and focuses on increasing autonomy, mostly through individual interactions. The Flexible ACT model underscores the importance of the content of the services delivered, yet it does not explicitly provide insight into the staging of interventions at the client level. Consequently, the extent to which teams can fulfill their intensive ACT (team-based crisis care) and less intensive non-ACT (treatment interventions through individual case management) functions throughout the service provision process is unclear. Given these considerations, this study examined the degree of model adherence and the content and staging of care according to the hourglass model provided by the teams.

By examining model fidelity and care content in Youth Flexible ACT teams, we aimed to contribute to the ongoing evaluation and advancement of evidence-based integrated care models that provide flexible levels of care for diverse problems. The current study aimed to provide a detailed description of the level of implementation of sixteen Youth Flexible ACT teams by investigating the following: 

1) Model fidelity: Do the participating teams perform work that aligns with the model? What elements are adequately deployed, and what are possible improvements?

2) Content and staging of care: What type of care is provided (over time)?

## Methods

### Study Design

This study was part of the Multicenter Youth Flexible ACT Study, a longitudinal observational prospective cohort study of 16 Youth Flexible ACT teams from 7 mental healthcare institutions throughout the Netherlands (Broersen et al., 2020a, 2020b). In the current study all sixteen teams were subjected to a single and formal audit. The teams were audited within 1.5 years (2016–2018). Teams not previously certified were audited as soon as possible, and other teams were audited as close to the certificate's expiration date as possible (certificates are valid for three years). Mental health workers were asked to complete a ‘content of care questionnaire’ 6 months after the baseline measurement of the cohort study (Broersen et al., 2023), with two follow-up measurements (12 and 18 months after baseline), to measure the content of treatment interventions provided in teams.

### Youth Flexible ACT Program

Flexible ACT is the Dutch adaptation and elaboration of ACT, which originated in the United States in the 1970s. Youth Flexible ACT, in which the family system plays a major part has important additional features beyond adult Flexible ACT. Youth Flexible ACT addresses the age-related developmental needs of children and young adults and supports the following developmental tasks: shaping changing relationships within the family, stimulating contact with peers, participating in education or work, and filling leisure time. It emphasizes increasing youth resilience by developing life skills appropriate to their life stages and transitions in collaboration with adolescents, families, and their (in)formal networks. This cooperation leads to shared goals aimed at improving the functioning in multiple life domains, encouraging adolescents to participate in the community, and enhancing their quality of life (Storm et al., 2013). 

A youth Flexible ACT team can be assembled from one institution or multiple organizations. In a ‘single organizational model’, the team consists (mostly) of mental health professionals from one organization and possibly some team members seconded from other organizations. In a ‘multi-organizational model’, multiple organizations deliver professionals to form a shared Youth Flexible ACT team (supported by one or multiple managers). A detailed description of the Youth Flexible ACT program is described in our case study paper (Broersen et al., 2022). 

### Youth Flexible ACT Client Population

Youth Flexible ACT can be delivered to children and adolescents (0–24 years of age) with wide-ranging, interrelated, and enduring psychiatric and social care needs. The Youth Flexible ACT population faces many problems in various areas of daily life, such as family stress, substance misuse, and/or problems with intellectual functioning (Broersen et al., 2020a, 2020b). They experience everyday difficulties, including problems with attending school, finding and keeping a job, peer relationships, housing, the legal system or police, and/or personal finance. Trauma and developmental, mood, and anxiety disorders are common in this population. The parents of these young people frequently endure parental stress and may also experience psychiatric issues themselves (Broersen et al., 2020a, 2020b; Vijverberg et al., 2018). This group faces complex and problematic challenges (e.g., risk of self-neglect, psychiatric decompensation, suicide, domestic violence, child abuse, or self-harm) with limited protective factors (e.g., adequate coping, employment, or daily structural activities and a support system). Regular office-based mental health services do not suffice, as these young people either drop out, are excluded from care services, or avoid care. See our baseline paper for a detailed description of the Youth Flexible ACT client group (Broersen et al., 2020a, 2020b).

### Model Fidelity 

#### Youth FACTs

The Youth Flexible ACT model fidelity scale (Youth FACTs) developed in 2014 was used to assess the implementation of the Youth Flexible ACT model (CCAF, 2014). The Youth FACTs is based on the adult FACT model fidelity scale. The scale is outlined in the Youth Flexible ACT model description (Hendriksen-Favier, 2013). Unlike the original adult FACT model fidelity scale, the Youth FACTs includes input from a family therapist, parent- and family counselors, and an employment and education specialist. It consists of 62 items (Online Resource 1) that address seven core elements: team structure (15 items); program process (12 items); assessment, treatment, and interventions (12 items); organization of services (11 items); community care (5 items); monitoring (3 items); and professional development (4 items).

#### Audit Procedure

In the Netherlands, the Centre for Certification and Flexible ACT (CCAF; https://ccaf.nl) conducts audits for Youth Flexible ACT. The CCAF trained professionals from the field as auditors to assess the Youth FACTs. Audits are performed by two individuals. One of the researchers in this study also received auditor training and was part of all auditor pairs. The audits conducted in this study are official and were performed in accordance with the standard procedures established by the CCAF. Two weeks before the audit, teams completed a background information form, providing details on team composition, caseload size, team admission rates and inpatient admissions. On the audit day, two auditors visited the team to collect information for completing the Youth FACTs through interviews, observations, and file reviews. During the audit, the auditors attended the daily team meeting (in which all clients receiving intensified team care were discussed using the digital FACT board), interviewed the team leader and at least two other team members, and joined a mental health worker on one or more client visits. Afterward, the auditors independently scored the scale, utilizing the manual for item score descriptions and calculations. Any discrepancies in scores were discussed between the auditors, and the final consensus scores were submitted to a senior auditor. After determining the final score, the team received the final report along with recommendations. The inter-rater agreement between pairs of independent assessors was high for the total scale scores (ICC = 0.91).

#### Youth FACTs Scoring

The auditors scored the items of the Youth FACTs on a 5-point rating scale ranging from 1 (minimal implementation) to 5 (maximal implementation). The total model fidelity score was obtained by taking the average across all items. According to the CCAF, scores ≤ 3.0 denote insufficient program implementation with no certificate, while scores > 3.0 and ≤ 3.3 indicate a temporary certificate for one year, with improvements to be made to obtain a final certificate. Scores > 3.3 and < 4.1 suggest ‘adequate implementation’ and thus can receive the certificate, while scores ≥ 4.1 are regarded as ‘optimal implementation’ (CCAF, 2014). 

### Content of Care

Mental health workers were requested to complete a ‘content of care questionnaire’ three times. This self-constructed ‘content of care questionnaire’ contained seven multiple-choice questions on the type of treatment and support, frequency of visits, and frequency of provided intensive ‘ACT’ care in the preceding six months (Online Resource 2). 

Among the cohort of 199 clients engaged in the Youth Flexible ACT study, mental health workers completed at least one ‘content of care questionnaire’ for 180 clients. Questionnaires were incomplete due to (premature) termination of care or due to mental health workers facing constraints in allocating sufficient time for questionnaire completion. A total of 115 first time-point (T1), 107 second time-point (T2), and 95 third time-point (T3) measurements were completed (Online Resource 3). For 36 clients only the first measurement was completed, for 49 clients two measurements were completed, and for 44 clients all three measurements were completed.

### Statistical Analyses

The researchers processed the audit and content of care data using IBM SPSS version 27.0. To examine whether content of care varied over time (staging of care), we applied Latent Growth Curve Analyses (LGCA) (Grimm et al., 2017) using the statistical package Mplus, version 7.2 (Muthén & Muthén, 1998–2015). All available data of the 180 clients were used. Missing data were handled using the Bayes estimator under the assumption that missing vales are Missing at Random (MAR) (Enders, 2011). A linear growth curve was assumed between outcomes and time (time was set as half-years: 0.5, 1 and 1.5), with intercept *i* (fixed at 0 for model identification purposes) and slope *s* (increase or decrease per time unit) as growth parameters. The linearity assumption will be supported if the fit of the linear model (expressed in Posterior Predictive P-value; PPP) is around 0.5 and at least > 0.10) (Cain & Zhang, 2019). For binary (Psychological intervention; Pharmacological treatment; Practical, supportive contacts; Psychodiagnostics; Family interventions; Scaled-up / intensified care; Inpatient admissions) and ordinal outcomes (Highest frequent level of care) probit regression was applied: the relationship between time and outcome is expressed in standard scores z, with most z-scores ranging between -3 and + 3. A change of one unit in time implies that the outcome variable (in terms of z) changes by the value of *s*.

## Results

### Model Fidelity

#### General Information

Between 2016–2018, model fidelity scores were established for 16 Youth Flexible ACT teams from 7 mental health institutions across the Netherlands. Among these, three teams served the catchment area of the second-largest city in the Netherlands, while the remaining teams covered mixed urban and rural areas. Most audits involved recertification (*n* = 9), with teams receiving initial certification in 2014. All teams started at least 2 years (median 4.5, range 2–12 years) before the current certification. Half of the teams (*n* = 8) followed a ‘single organizational model’ and the other half adhered to a ‘multi-organizational model’. On average, the teams consisted of 10.3 team members (range 6—16) with an average of 6.6 FTE caregivers (*SD* = 2.5; range 2.9—11.9). The average number of clients per team was 74.8 (*SD* = 30.2; range 25—148). This implies an average staff-client ratio of 1:11.3. Youth Flexible ACT teams included an average of 20 clients (range 5—37 clients per team), during the 6-month period preceding the audit. At the time of the audit, 8.4% of the clients (range 1—26 clients per team) were admitted to a (general) psychiatric hospital unit or temporarily resided elsewhere, and an additional 0.4% were in detention.

#### Model Fidelity Scores 

**Fig. 1:**
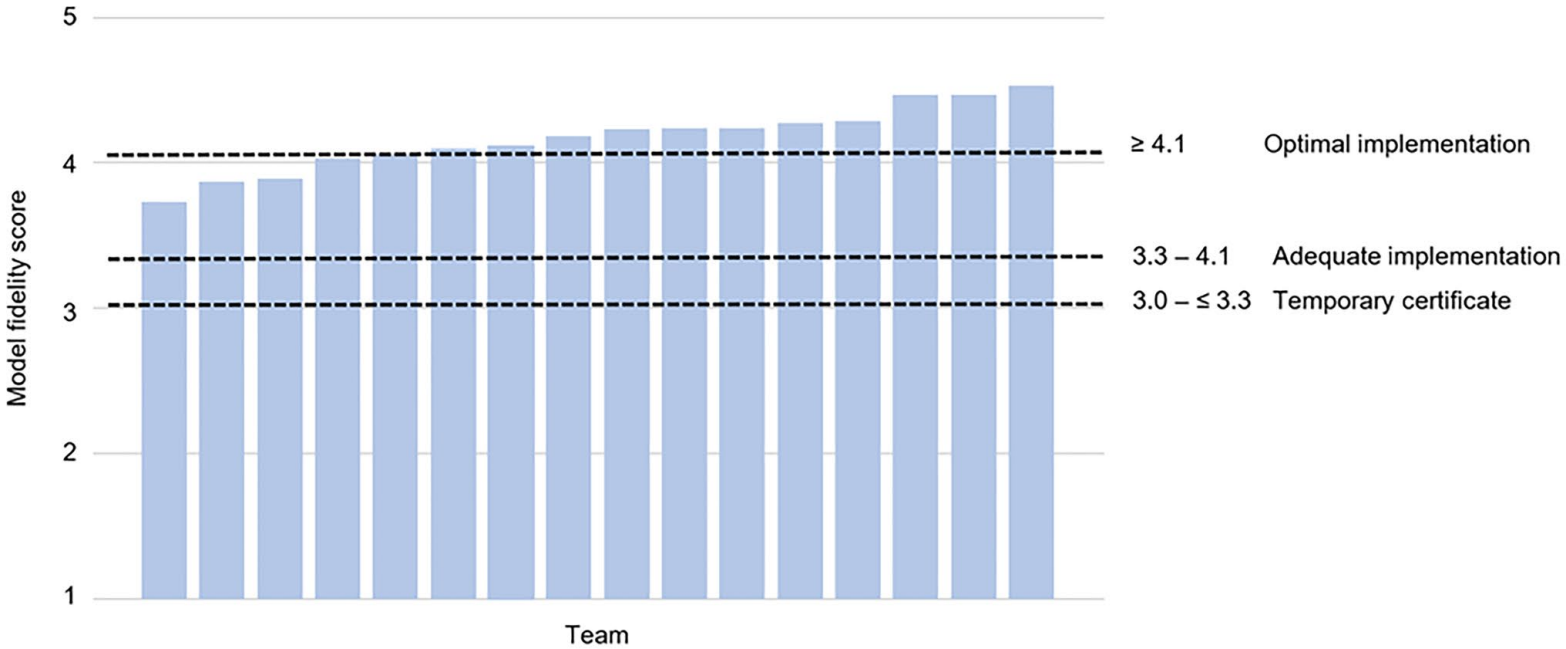
Total Model Fidelity Scores of the 16 Youth Flexible ACT Teams

The mean overall model fidelity score was 4.17 (*SD* = 0.22), ranging from 3.73 to 4.53 (Online Resource 1). Twelve teams received the ‘optimal implementation’ score (≥ 4.1), and four teams attained ‘adequate implementation’ (score between 3.3 and 4.1) (Fig. 1).

To qualify the results at item and subscale levels, we used the same four score brackets defined by CCAF at a total score level and used the following terms: ‘insufficient’, ‘moderate’, ‘adequate’, and ‘optimal’ (see Table 1). Across teams, 5 items showed insufficient implementation, 2 indicated moderate implementation, 15 showed adequate implementation, and 40 showed optimal implementation.

**Table 1:** Youth FACTs Subscale and Item Scores Grouped Into Four Score Brackets

	Insufficient implementation (score ≤ 3.0)	Moderate implementation (3.0 < score ≤ 3.3)	Adequate implementation (3.3 < score < 4.1)	Optimal implementation (score ≥ 4.1)
Team structure (*M* = 4.04, *SD* = 0.29)	Peer support worker, Employment and education specialist		Team member employment, Family therapist, Social worker and Psychiatric nurses, Family and parental counseling expertise, Reflection of caseload	Small caseload, Staff capacity, Child & Adolescent psychiatrist, Child & Adolescent psychologist, Total FTE for three treatment specialists, Case manager, Addiction expertise, Expertise relating to MID
Program process (*M* = 4.18, *SD* = 0.39)	Procedure discharge FACT-board, Frequency of contact – ACT		Team approach – non-ACT, FACT-board admission procedure	Team approach – ACT, Daily team meeting, Multidisciplinary team meeting, Treatment plan – disciplines, Treatment plan – clients, FACT-board admission criteria, Frequency of contact – non-ACT, Modern communication tools
Assessment, treatment, and interventions (*M* = 4.27, *SD* = 0.33)			Psychoeducation, Family interventions, Integrated Dual Disorder Treatment, Employment and Education programs	(Process)diagnostics, Multidisciplinary practical care services, Individual treatment plan, Individual crisis plan, Individual developmental and recovery-oriented goals, Copy treatment plan, Medication management, Psychological treatment
Organization of services (*M* = 4.45, *SD* = 0.45)		,	Restorative Time-Out	Admission criteria, Waiting list, 24-h accessibility and crisis, Risk assessment tools, Responsibility for hospital admission, Visits during admission, Responsibility for hospital discharge, Gradual transfer, Discharge from FACT, No dropout
Community care (*M* = 4.62, *SD* = 0.36)				Outreach services, Systematic involvement of external partners, Cooperation with a formal support system, Cooperation with an informal support system – ACT, Cooperation with an informal support system – non-ACT
Monitoring (*M* = 3.05, *SD* = 0.63)	Use of ROM	Routine Outcome Monitoring (ROM) – instruments	FACT quality improvement	
Professional development (*M* = 3.89, *SD* = 0.62)		Structural focus on developmental- and recovery-oriented practice	Reflective practice, Team spirit	Training FACT- and related subjects

*Note.* MID = Mild Intellectual Disability; FACT = Flexible Assertive Community Treatment; ACT = Assertive Community Treatment

 The subscale ‘community care’ achieved the highest score of 4.62, with all items in the optimal range. This implies that the teams possessed an extensive professional network with the web of community resources. The teams actively maintained structural contacts with schools, municipalities, youth services, youth mental health organizations, adult psychiatry, assisted living projects, and work-study companies. Additionally, the teams actively involved the informal support system during both intensive and less intensive care periods. The organization of the program services (subscale ‘organization of services’; e.g., entry and exit protocols for FACT, risk assessment procedures, responsibilities regarding inpatient admissions; a score of 4.45) and the overall basic Flexible ACT procedures (subscale ‘program process’ e.g., shared caseload, daily team meetings, multidisciplinary collaboration; a score of 4.18) were also implemented optimally on average. The teams could flexibly adapt the level of care within the team and across the whole continuum of care. Yet, the contact frequency during scaled-up/intensified care was insufficient in 7 teams (≤ 2 face-to-face appointments per week; > 3.0 face-to-face appointments per week is scored as adequate). Additionally, not all patient admission and discharge criteria from the digital FACT board were met systematically (7 and 13 teams received insufficient scores, respectively). Clients and their support systems were not consistently informed, and client satisfaction was not systematically assessed after the intensified care period. Although the crisis and treatment plan were modified when necessary, they were not routinely updated with all clients.

The subscale ‘assessment, treatment, and interventions’ (score of 4.27) was implemented optimally across teams, and the subscale ‘team structure’ (score of 4.04) was implemented adequately. This indicates that the teams offered diverse diagnostics and specialized treatment options. Yet, several items assessing the involvement of specific disciplines and the availability of treatment interventions (peer support worker, employment and education specialist – and programs, family interventions, Integrated Dual Disorder Treatment) scored below the optimum, suggesting room for improvement. Especially the implementation of the peer support worker and employment and education specialist was insufficient. Only three teams had a peer support worker. Also, nine teams scored insufficient on the item 'employment and education specialist'. In these teams, multiple team members assumed the employment and education specialist roles without specific training, as no specific employment and education specialist was appointed. Consequently, these teams demonstrated inadequate implementation of employment and education programs (ten teams scored insufficient on the ‘employment and education programs’ item). While teams actively supported and guided clients in pursuing or resuming (mostly) education and (sometimes) employment, they did not use effective interventions. Notably, only two teams had staff members who had received training in applying the Individual Placement and Support (IPS) model. ‘Family interventions’ were implemented adequately across teams, yet insufficient scores were noted for seven teams. Among these, three teams lacked a formally registered family therapist, while four teams received insufficient scores due to the family therapist being underemployed, serving fewer than 30% of families. Furthermore, seven teams scored insufficient on the ‘Integrated Dual Disorder Treatment (IDDT)’ item. Among these teams, three teams could not designate an addiction expert who met the model criteria (no specific work experience and/or education), which prevented the complete execution of the IDDT model, compromising collaboration with an addiction care institution, for example. In the case of the remaining four teams, despite having a team member with expertise in addiction, the complete model criteria for IDDT were not obtained. Teams did not apply the stages of behavior change clearly and methodologically and were often not trained in motivational interviewing. 

The subscale professional development’ was implemented adequately on average across teams (score of 3.89). This means that teams paid attention to their professional development by following training courses in Flexible ACT and/or related topics. Teams were attentive to clients' developmental, empowerment, and recovery processes during the entire care process (item ‘Structural focus on developmental- and recovery-oriented practice’), which was particularly reflected in concrete actions at the client level (e.g., joining a sports club, taking the step to go back to school, choosing a different place to live). Nevertheless, nine teams obtained insufficient scores. This was mainly due to 1) the low number of specific interventions deployed (e.g., Wellness Recovery Action Plan) and 2) the observation that clients’ and families’ self-capability and strengths received too little attention during the daily team meetings and in the goals described in the clients’ treatment plans. 'Reflective practice' was insufficient in five teams with no regular intervision meetings. Teams attributed this to the high workload, leading to the intervision meetings being dropped first. 

The subscale ‘monitoring’ achieved the lowest score (score of 3.05; moderate implementation). Specifically, while standardized measuring instruments were used annually to measure the functioning of the clients (item ‘Routine Outcome Monitoring (ROM) – instruments’), the results were underutilized at the individual client level and the team level (item ‘use of ROM’).

### Content of Care

The model fidelity results presented above provide insight into the care organization. The next section describes the content of care offered to our Youth Flexible ACT sample. A comprehensive overview of the results can be found in Table 2.

**Table 2:** Content of Care Data of the 16 Youth Flexible ACT teams

Content of care	T1: 0–6 mths in care (*n* = 115)	T2: 6–12 mths in care (*n* = 107)	T3: 12–18 mths in care (*n* = 95)	Total (*n* = 180)^a^	Slope	*p*	PPP
Psychological intervention (individual)	103 (89.6%)	87 (81.3%)	71 (74.7%)	160 (88.9%)	− 54	.082	.532
- Psychoeducation	91 (79.1%)	69 (64.5%)	55 (57.9%)	152 (84.4%)			
- CBT	37 (32.2%)	30 (28%)	25 (26.3%)	65 (36.1%)			
- EMDR	19 (16.5%)	9 (8.4%)	13 (13.7%)	33 (18.3%)			
- Substance abuse treatment	5 (4.4%)	5 (4.7%)	5 (5.3%)	10 (5.6%)			
- PMT	2 (1.7%)	3 (2.8%)	2 (2.1%)	5 (2.8%)			
- Schema therapy	1 (0.9%)	7 (6.5%)	5 (5.3%)	9 (5.0%)			
- ERT	3 (2.6%)	5 (4.7%)	7 (7.4%)	13 (7.2%)			
- ART	3 (2.6%)	4 (3.7%)	3 (3.2%)	8 (4.4%)			
Psychological intervention (group)	6 (5.2%)	7 (6.5%)	10 (10.5%)	16 (8.9%)			
Pharmacological treatment	59 (51.3%)	52 (48.6%)	46 (48.4%)	101 (56.1%)	− .05	.862	.492
Consults with a peer support worker	5 (4.4%)	6 (5.6%)	3 (3.2%)	10 (5.6%)			
Practical, supportive contacts	98 (85.2%)	86 (80.4%)	81 (85.3%)	153 (85%)	.75	.160	.494
- Finances	24 (20.9%)	12 (11.2%)	20 (21.1%)	42 (23.3%)			
- Housing	39 (33.9%)	30 (28%)	32 (33.7%)	68 (37.8%)			
-School/work/daytime activities	64 (55.7%)	44 (41.1%)	56 (59%)	111 (61.7%)			
Psychodiagnostics	45 (39.1%)	26 (24.3%)	14 (14.7%)	70 (38.9%)	− 1.70	.000	.507
- Psychodiagnostic assessment	17 (14.7%)	16 (15%)	9 (9.5%)	34 (18.9%)			
- Data file review	23 (20%)	9 (8.4%)	3 (3.2%)	32 (17.8%)			
- Developmental- and Social history assessments	16 (13.9%)	5 (4.7%)	0 (0%)	19 (10.6%)			
Family interventions	76 (66.1%)	60 (56.1%)	58 (61.1%)	122 (67.8%)	− .08	.816	.501
- Family systemic therapy	24 (20.9%)	17 (15.9%)	23 (24.2%)	45 (25.7%)			
- Parental counseling	69 (60%)	49 (45.8%)	49 (51.6%)	109 (60.6%)			
Scaled-up / intensified care	41 (35.7%)	34 (31.8%)	29 (30.5%)	75 (41.7%)	− .20	.630	.494
- increase in symptoms	36 (31.3%)	29 (27.1%)	24 (25.3%)	68 (37.8%)			
- care-avoiding behavior	12 (10.4%)	5 (4.7%)	7 (7.4%)	22 (12.2%)			
- psychiatric inpatient admission	5 (4.4%)	5 (4.7%)	3 (3.2%)	11 (6.1%)			
- change of treatment	4 (3.5%)	5 (4.7%)	8 (8.4%)	16 (8.9%)			
- life events	6 (5.2%)	6 (5.6%)	9 (9.5%)	18 (10.0%)			
Highest frequent level of care	6 (5.2%)	14 (13.1%)	19 (20%)	15 (8.3%)	− .68	.002	.488
- <1 appointment every 14 days	12 (10.4%)	10 (9.4%)	9 (9.5%)	15 (8.3%)			
- 1 appointment every 14 days	39 (33.9%)	34 (31.8%)	30 (31.6%)	53 (29.4%)			
- 1 appointment every 7 days	53 (46.1%)	46 (43.0%)	35 (36.8%)	89 (49.4%)			
- Multiple appointments in the week	5 (4.4%)	3 (2.8%)	2 (2.1%)	8 (4.4%)			
- Daily appointments							
Inpatient admissions	17 (14.7%)	14 (13.1%)	7 (7.4%)	7 (7.4%)	− 1.22	.005	.497
- Planned	14 (12.2%)	10 (9.4%)	5 (5.2%)	5 (5.2%)			
- Not planned	6 (5.2%)	4 (3.8%)	2 (2.1%)	2 (2.1%)			

*Note.* CBT = Cognitive Behavioral Therapy, EMDR = Eye Movement Desensitization and Reprocessing, PMT = Psychomotor Therapy, ERT = Emotion Regulation Therapy, ART = Aggression Regulation Therapy; PPP = Posterior Predictive P-value

^a^Total number of clients for whom at least one content of care questionnaire was completed by a mental health care worker at either T1, T2, of T3

 A substantial proportion of clients (88.9%) received an individual psychological intervention, including psychoeducation (84.4%), cognitive behavioral therapy (36.1%), eye movement desensitization and reprocessing (18.3%), and emotion regulation therapy (7.2%). Pharmacological treatment was administered in 56.1% of cases. Substance abuse treatment and the deployment of a peer support worker were provided in 5.6% of cases. In addition, a large group (85%) received practical support, including personal finance (23.3%), housing (37.8%), or school/work/daily activities (61.7%). Psychodiagnostics, such as psychodiagnostic assessments (18.9%) in addition to developmental and social history assessments (10.6%) and data file reviews (17.8%), was utilized with about 40% of the clients.

The Youth Flexible ACT client population often faces problems in the family. This was indeed reflected by the high ratio of family interventions (67.8%), including parental counseling (60.6%) and family systemic therapy (25.7%). Teams that did not have (adequate) experts in family relations and/or system therapists (according to the model guidelines; score 1 or 2) also offered fewer care services in these areas compared to the teams that scored sufficiently on these items (parental counseling 59.5% vs. 77.2% and family systemic therapy 10.3% vs. 31.9%). 

The highest average frequency of care involved daily contact, which was required for 4.4% of clients. Half of the clients (49.4%) had multiple appointments over seven days. Around one-third (29.4%) of clients had one appointment over seven days, 8.3% of clients had one appointment over 14 days, and 8.3% of clients had less than one appointment every 14 days. For 41.7% of clients, the care level was scaled-up / intensified throughout care, mostly due to increasing symptoms. Furthermore, 27 out of 180 clients (15%) were admitted to a (general) psychiatric hospital unit during the period of care.

#### Stages of Care 

Eight linear models were tested with PPP-values between 0.488 and 0.532, supporting the linear assumption between time and outcome. Regarding the content of care over time, the results showed that psychological interventions (*s* = -0.54, *p* = 0.082), pharmacological treatment (*s* = -0.05, *p* = 0.862) practical, supportive contacts (*s* = 0.75, *p* = 0.160), family interventions (*s* = -0.0.8, *p* = 0.816), scaled-up / intensified care (*s* = -0.20, *p* = 0.630) did not show a significant decrease over time (Table 2). Psychodiagnostic assessments (*s* = -1.70, *p* =  < 0.001), highest frequent level of care (*s* = -0.68, *p* = 0.002) and inpatient admissions decreased significantly over time (*s* = -1.22, *p* = 0.005), indicating increased stability in functioning.

## Discussion

### Model Implementation 

All teams implemented the Youth Flexible ACT model adequately to optimally, achieving a high-quality organization of care. Strengths included community care services, collaboration with formal and informal support systems, and a diverse range of specialized psychological interventions. The basic Flexible ACT procedures and organization of various services were implemented appropriately. Professionals from diverse fields, such as psychologists, psychiatrists, and psychiatric nurses, were well integrated. However, some teams had difficulties integrating disciplines emphasizing personal and social recovery, particularly peer support workers and employment and education specialists. 

#### Obstacles in Disciplinary Implementation 

Implementing the required disciplines poses multiple challenges. First of all, funding is a crucial factor. For instance, the lack of reimbursement for deploying peer support workers in the Dutch healthcare system probably resulted in their presence being limited to only three teams. This limitation is unfortunate, as peer support workers could greatly improve the provision of youth-friendly, recovery-oriented services that prioritize clients’ perspectives (De Beer et al., 2022; Hiller-Venegas et al., 2022) and improve treatment engagement (Ojeda et al., 2021). Another important aspect to recognize is the readiness of the work environment to accommodate peer support workers. Achieving this readiness necessitates a cultural shift within mental health organizations from the conventional medical model (prioritizing clinical expertise within a hierarchical structure) to a recovery-oriented system (wherein peer support workers coexist with clinical expertise; De Beer et al., 2022). Some teams struggled with this issue. 

Second, certain teams adopted a policy of using (instead of integrating) experts from other sections of the organization to save costs, yet this practice was challenging to implement effectively. An example of this was evident in the deployment of a family therapist across multiple teams who was able to serve fewer than 30% of families in the respective Flexible ACT teams. This pattern reflects the integration of disciplines without specific roles and with multiple team members assuming these roles without requisite training. For example, taking on the roles of the 'employment and education specialist' and the 'addiction expert' led to shortcomings in the implementation and execution of protocolled interventions (such as IPS and IDDT). These findings also likely have implications for positive client outcomes. Our observational cohort study revealed an overall enhancement in clients’ attitudes toward education and work. However, this positive shift did not result in more adolescents accessing appropriate education or employment opportunities during the 18 months of Youth Flexible ACT (Broersen et al., 2023). A mental healthcare worker with a broad network and specialized training (such as IPS, preferably enhanced with cognitive remediation; Killackey et al., 2019; Erickson, 2021; Van Duin et al., 2021) may improve outcomes in this domain.

Furthermore, especially within multi-agency models, the organization and coordination of interventions posed substantial challenges stemming from healthcare providers' need to distribute responsibilities (alongside associated financial aspects). Moreover, staff shortages in the healthcare sector were a limiting factor in filling vacancies.

In sum, opportunities for quality improvement exist within personal and social recovery domains, particularly concerning the integration of specialized disciplines and protocolled interventions.

Clearly defining roles and designating staff members dedicated to these functional areas of recovery can enhance the efficiency of care.

### Content of Care

#### Non-ACT function

Overall, the ‘content of care’ findings demonstrate that teams could conduct psychodiagnostic and psychological interventions, despite the crisis sensitivity of the client population. Moreover, the interventions applied match well with the needs of the Youth Flexible ACT population (Broersen et al., 2020a, 2020b). For instance, the population reported problems with the family situation, and family interventions were deployed in 67.8% of cases. However, some interventions were employed to a lesser extent than anticipated. First, substance abuse treatment, for instance, was only administered in 5.6% of cases, even though teams estimated that substance abuse issues accounted for approximately 25% of the caseload (range: 4%—50%). In addition, the observation that addiction experts were well represented in the teams does not match the limited utilization of substance abuse treatments, as well as the insufficient implementation of IDDT in approximately half of the teams. It is possible that substance abuse issues were addressed within other or external treatments, and as such were underreported in our study. Moreover, the incomplete implementation of the IDDT model may be related to insufficient training of the team or inadequate collaboration with addiction care institutions. Second, although a key role in the Youth-Flexible ACT model, the peer support worker was only involved in 5.6% of the cases, and was present in only three teams. Third, psychodiagnostics had been performed on 40% of the clients. While this percentage might appear modest, it is noteworthy that this item primarily referred to (a form of) psychodiagnostic assessment that was conducted with (most) clients before Flexible ACT care (Broersen et al., 2020a, 2020b). Indeed, according to model fidelity data teams scored high on the process diagnostics item, highlighting the ongoing and iterative nature of diagnostic procedures throughout care provision. 

#### ACT function

Both model fidelity data and content of care data indicate that teams provided intensive, team-based crisis care when required (ACT function). This is evident in aspects related to team approach (shared caseload), daily team meetings, digital FACT board procedure, and in offering multiple appointments per week when needed. The content of care data showed that over the past 18 months, there was at least a period where clients received multiple (≥ 2) appointments per week. Yet, the model fidelity data showed that frequency of contact was not sufficient (< 3 appointments per week) throughout periods of ACT care. In future research, it would be interesting to explore why intensification of care can be achieved incidentally but not consistently. It may be possible that the caseload is too high for members of the team to be able to provide the required frequency of contact. It should also be noted that the frequency of contact was averaged across all clients scaled-up on the digital FACT board, assuming these clients all received intensified care. However, we encountered anecdotal evidence of clients having been put on the FACT board with the purpose of monitoring their condition in the team daily, without requiring acute intensified care. This may suggest that teams require more degrees of treatment intensity than a dichotomy between ACT and non-ACT.

#### Stages of Care

As stated in the introduction, although the Flexible ACT framework underscores the significance of the content of the services delivered, it does not explicitly provide insight into the staging of interventions at the client level. The hourglass model (Van Veldhuizen & Bähler, 2013) is an important part of the Flexible ACT framework and distinguishes three stages of care: stabilization, treatment, and recovery. According to this model, the initial stage when clients start Flexible ACT centers on stabilization (Stage 1). The emphasis shifts to treatment once the primary crisis abates (Stage 2). As symptoms start to diminish, full focus can be directed toward recovery (Stage 3). The hourglass graphic represents a process that proceeds serially, yet the designers of the hourglass model also describe that the stages are not sharply delineated and that recovery (Stage 3) overlaps with previous stages. Our data showed that crisis and stabilization (Stage 1) and treatment (Stage 2) occur parallel amongst the youth population. Recovery (Stage 3) was observed throughout the care process, consistent with the hourglass model. The case manager continually supported recovery by focusing on developmental objectives throughout all stages of care.

Contrary to what the hourglass model suggests, the stages within Youth Flexible ACT care do not follow a serial process but rather a dynamic one in which different aspects of care (ACT, treatment, and recovery) can be addressed simultaneously. In our case study, we presented a comprehensive portrayal of a client, demonstrating the simultaneous execution of different stages of care (Broersen et al., 2022). The client successfully engaged in care, ultimately completing trauma treatment despite facing diverse personal challenges that posed obstacles to the treatment trajectory. Collaboration among diverse team members and consistent utilization of practical, supportive contacts were essential in maintaining the treatment trajectory.

### Exiting Flexible ACT 

The content of care data implied that 18 months of Flexible ACT care may not be sufficient for a substantial group of clients. The average length of Flexible ACT involvement in this study was 21.0 months (*SD* = 9.7; range 3–40 months), yet some adolescents (*n* = 113) still received Youth Flexible ACT care after the data collection period. To get a complete picture of the treatment duration, we requested treatment duration data from two participating mental health organizations, encompassing the period from the initiation of Flexible ACT in 2014 to 2019. The results generally aligned with our study sample: 17.2 months (*SD* = 12.5; range 3–77 months, *n* = 807) and 20.9 months (*SD* = 13.8; range 3–74 months, *n* = 1239) (clients that dropped out in the first two months were excluded). This suggests that, although an average duration of 18 months in Flexible ACT is generally suitable, substantial individual variation exists. The data prompted questions, leading us to consider the possibility of three (non-exhaustive) groups 1) A group that no longer requires the level of intensity of Flexible ACT care after 1–1.5 years of goal-oriented treatment, 2) a smaller group that requires an extended emphasis on initial crisis management, thereby prolonging the dependence on Flexible ACT to some degree, and 3) another group requiring prolonged and intensive support. This indicates the need to tailor the duration of Flexible ACT and the exit strategy to the circumstances, progress, and evolving needs of the client. 

### Study Limitations

First, the mental health workers did not consistently complete the ‘content of care’ questionnaires for all participating clients across all measurement points. In our analyses, we included all available data rather than solely focusing on the data from the participants for whom all three measurements were available. Moreover, a noteworthy number of participants exited care during the study period, disrupting the continuous monitoring of care trajectories, potentially resulting in an incomplete depiction of the nuanced shifts in care content that may have occurred over time. 

Next, we conclude that teams are proficient in delivering Non-ACT and ACT functions as part of their service provision. However, we did not assess the quality of provided services at the client level, such as the therapeutic relation and adherence to treatment protocols. The presence of a healthcare psychologist (as prescribed according to the model) does not necessarily guarantee that the interventions were genuinely evidence-based or aligned with guideline-driven treatments for conditions like trauma, depression, and psychosis. Achieving a high score is feasible even if the interventions do not strictly adhere to the guidelines. Combined with the audit scores revealing inconsistencies in the successful implementation of interventions or specific protocolled models (e.g., IPS, IDDT), we infer that the quality of treatment could be further enhanced.

### Youth FACTs Limitations

Both teams and auditors perceived the Youth FACTs as a quantitative checklist with clearly yet narrowly defined, somewhat rigid criteria. These criteria provided little flexibility to adapt to variations in team caseloads or local context. Teams utilized more liberal approaches to achieve their objectives, as was apparent from our results on admitting and discharging clients on the digital FACT board. In addition, additional flexibility in the way that contact frequency is measured would better match the world of youths, by not only regarding face-to-face contact, but also include contact via phone calls or chat messages. Consequently, rigidly adhering to the item criteria during scoring may not consistently capture the nuanced subtleties of real-world practice or encourage teams to incorporate innovations and practices.

On the one hand, scoring should be based on criteria that encapsulate the essential elements of the model and underlying principles rather than on the exact wording of the fidelity scale (Kroon & Bähler, 2015). On the other hand, adhering to the model guidelines offers substantial advantages (Bond et al., 2000). Clearly defined model criteria for starting and developing teams serve as a valuable guiding framework for initiating their operations. As caseloads expand, the diversity of clients' needs grows accordingly. Adhering to the model becomes essential in such circumstances to uphold a structured overview. 

Overall, the quality framework remains an evolving process, continuously shaped by the ongoing interchange between theory and practical application (Teague et al., 2012). In the context of adult Flexible ACT, these constraints prompted the development of a modified Flexible ACT model fidelity scale in 2017 (Westen et al., 2021).) This revised scale incorporates qualitative and quantitative elements to improve implementation beyond a checklist approach. A novel youth Flexible ACT scale was also created in 2020, known as FACTs-Youth 2020 (CCAF, 2020). We collected our data before the release of this scale.

## Conclusion

Our study showed that the Youth Flexible ACT teams have successfully translated care from the theoretical Flexible ACT framework into practice. Further room for improvement lies in incorporating professionals in specialized disciplines, including peer support workers and employment and education specialists, in personal and social recovery and specific protocolled interventions, such as IDDT, systemic interventions, and IPS. Regarding the content of care, the teams adeptly delivered different aspects of care, both intensive crisis care and treatment and recovery interventions, despite the population's varying dynamics and care needs.

### Supplementary Information

Below is the link to the electronic supplementary material.

Supplementary file1 (PDF 254 KB)

Supplementary file2 (PDF 229 KB)

Supplementary file3 (PDF 84 KB)
