# Supplementary material for: Examining Youth Flexible ACT Model Implementation in the Netherlands
Source: Community Ment Health J. 2024 Mar 22;60(6):1081–93. doi: 10.1007/s10597-024-01260-z (PMC11199218; doi:10.1007/s10597-024-01260-z)
Supplement: Supplementary file 1 — Supplementary file1 (PDF 254 KB) [file 10597_2024_1260_MOESM1_ESM.pdf]

Online Resource 1

Item, Subscale and Total Model Fidelity Scores of the 16 Youth Flexible ACT Teams

	Team 1	Team 2	Team 3	Team 4	Team 5	Team 6	Team 7	Team 8	Team 9	Team 10	Team 11	Team 12	Team 13	Team 14	Team 15	Team 16	*M*	*SD*
**Team structure**																		
1. Small caseload	5	4	5	5	5	4	5	5	5	5	5	5	5	5	5	5	4.88	0.34
2. Staff capacity	5	3	5	5	5	5	5	5	5	5	5	5	4	5	5	5	4.81	0.54
3. Team member employment ‘single agency model’	5		4	4	5		2			3			4	4			3.62	0.89
Team member employment ‘multi agency model’		3				3		4	3		4	2			4	4		
4. Child & Adolescent psychiatrist	3	5	5	5	5	4	5	5	2	5	5	5	3	5	3	5	4.38	1.03
5. Family therapist	4	5	1	1	1	5	5	2	5	5	4	5	5	3	5	5	3.81	1.64
6. Child & Adolescent psychologist	5	5	5	5	5	5	5	5	5	5	5	5	5	5	5	5	5.00	0.00
7. Total FTE for psychiatrist, psychologist and family therapist	3	5	5	4	5	5	5	5	5	5	3	4	4	4	4	5	4.63	0.72
8. Peer support worker	5	1	1	1	1	1	1	1	1	1	1	1	5	1	5	1	1.75	1.61
9. Social worker and Psychiatric nurses	5	2	5	3	4	5	5	4	2	5	5	2	2	5	4	2	3.75	1.34
10. Case manager	5	5	5	5	5	5	5	5	4	5	5	5	5	5	5	5	4.94	0.25
11. Addiction expertise	5	5	5	5	1	3	3	5	5	4	5	1	5	5	5	5	4.19	1.42
12. Employment and education specialist	2	1	1	4	1	5	5	2	5	4	5	1	2	1	5	1	2.81	1.80
13. Expertise relating to MID	5	5	1	5	5	5	1	5	5	3	5	5	5	1	5	5	4.13	1.63
14. Family and parental counseling expertise	2	4	5	3	5	5	5	1	4	5	5	5	2	2	5	5	3.94	1.44
15. Reflection of caseload	5	2	4	5	4	4	5	4	5	2	5	4	4	2	4	5	4.00	1.10
Average score Team structure	*4.27*	*3.67*	*3.80*	*4.00*	*3.80*	*4.27*	*4.13*	*3.87*	*4.07*	*4.13*	*4.47*	*3.73*	*4.07*	*3.60*	*4.60*	*4.20*	*4.04*	*0.29*

**Program process**																		
16. Team approach – non-ACT	5	2	5	4	5	4	3	4	5	5	2	2	5	3	5	2	3.81	1.28
17. Team approach – ACT	4	5	3	5	5	4	5	5	4	5	5	5	5	5	5	5	4.75	0.58
18. Daily team meeting	5	4	5	5	4	5	2	4	5	2	4	3	5	4	4	5	4.13	1.03
19. Multidisciplinary team meeting	5	5	5	5	5	5	5	5	5	5	4	5	5	5	5	5	4.94	0.25
20. Treatment plan – disciplines	5	5	5	5	5	5	5	5	1	5	5	5	5	5	5	5	4.75	1.00
21. Treatment plan – clients	5	5	5	5	5	5	3	5	5	5	5	5	5	5	5	4	4.81	0.54
22. FACT-board admission criteria	5	1	4	5	5	5	5	5	5	4	5	1	5	5	5	4	4.31	1.35
23. FACT-board admission procedure	5	2	2	5	5	2	4	5	3	4	5	1	4	3	4	3	3.56	1.32
24. Procedure discharge FACT-board	5	3	1	5	2	1	3	2	3	1	3	1	5	3	2	2	2.63	1.41
25. Frequency of contact – ACT	3	2	1	3	2	2	2	4	2	3	3	3	2	3	3	3	2.56	0.73
26. Frequency of contact – non-ACT	5	4	5	5	5	5	5	5	5	5	5	5	5	5	5	5	4.94	0.25
27. Modern communication tools	4	5	5	5	5	5	5	5	5	5	5	5	5	5	5	5	4.94	0.25
Average score Program process	*4.67*	*3.58*	*3.83*	*4.75*	*4.42*	*4.00*	*3.92*	*4.50*	*4.08*	*4.08*	*4.25*	*3.42*	*4.67*	*4.25*	*4.42*	*4.00*	*4.18*	*0.39*
**Assessment, treatment, and interventions**																		
28. (Process)diagnostics	5	4	4	4	5	4	4	4	5	5	5	5	4	5	5	4	4.50	0.52
29. Multidisciplinary practical care services	5	4	5	5	5	5	5	4	4	4	5	5	5	5	5	5	4.75	0.45
30. Individual treatment plan	5	3	5	5	5	5	5	4	4	4	4	5	5	4	5	5	4.56	0.63
31. Individual crisis plan	5	4	5	5	5	2	5	5	4	4	5	4	4	5	5	2	4.31	1.01
32. Individual developmental and recovery-oriented goals	5	5	4	5	5	5	4	5	5	4	5	5	5	5	4	2	4.56	0.81
33. Copy treatment plan	5	5	5	5	5	5	5	5	5	5	5	5	5	5	5	1	4.75	1.00

34. Medication management	5	5	5	5	5	5	5	4	5	4	5	5	5	5	4	3	4.69	0.60
35. Psychoeducation	5	5	3	3	4	3	5	3	4	5	4	3	3	3	5	3	3.81	0.91
36. Psychological treatment	5	5	4	5	4	5	5	5	5	5	5	5	5	5	5	5	4.88	0.34
37. Family interventions	3	5	2	2	2	4	5	2	5	4	4	5	5	5	2	3	3.63	1.31
38. Integrated Dual Disorder Treatment	5	4	3	4	4	2	2	4	5	2	4	2	4	3	2	4	3.37	1.09
39. Employment and Education programs	3	4	3	4	3	3	4	2	3	3	4	3	4	3	5	3	3.38	0.72
Average score Diagnostics, treatment and interventions	*4.67*	*4.42*	*4.00*	*4.33*	*4.33*	*4.00*	*4.50*	*3.92*	*4.50*	*4.08*	*4.58*	*4.33*	*4.50*	*4.42*	*4.33*	*3.33*	*4.27*	*0.33*
**Organization of services**																		
40. Admission criteria	5	4	2	5	3	5	5	5	5	5	5	5	5	5	5	5	4.63	0.89
41. Waiting list	5	5	3	4	5	2	4	3	5	4	5	5	5	4	5	5	4.31	0.95
42. 24-hour accessibility and crisis	4	5	4	5	4	4	4	4	5	4	5	4	5	5	4	4	4.38	0.50
43. Risk assessment tools	5	4	4	4	4	4	5	4	4	5	5	4	5	5	5	4	4.44	0.51
44. Responsibility for hospital admission	5	4	4	4	4	4	5	4	5	5	5	5	5	5	5	5	4.63	0.50
45. Restorative Time-Out[1]	5	3	1	3	1	5	5	3	5	5	5	3	1	4	3	5	3.56	1.55
46. Visits during admission	5	3	3	3	3	3	5	5	5	5	5	3	5	5	5	5	4.25	1.00
47. Responsibility for hospital discharge	5	5	5	4	5	3	5	3	5	5	5	4	5	5	5	5	4.63	0.72
48. Gradual transfer	5	5	5	5	5	5	5	5	5	4	5	5	5	5	5	5	4.94	0.25
49. Discharge from FACT	5	4	5	5	5	5	5	5	5	5	5	5	5	5	2	4	4.69	0.79
50. No drop-out	5	5	2	3	2	5	5	5	5	5	5	4	5	5	4	5	4.38	1.09
Average score Organization of services	*4.91*	*4.27*	*3.45*	*4.09*	*3.73*	*4.27*	*4.82*	*4.18*	*4.91*	*4.73*	*5.00*	*4.36*	*4.64*	*4.82*	*4.36*	*4.73*	*4.45*	*0.45*
**Community care**																		
51. Outreach services	5	3	4	4	5	5	5	5	5	5	5	3	5	5	4	5	4.56	0.73

52. Systematic involvement of external partners	5	5	5	5	5	5	5	5	5	3	3	4	5	5	5	5	4.69	0.70
53. Cooperation with a formal support system	4	4	4	4	5	5	4	5	5	5	4	5	5	5	5	4	4.56	0.51
54. Cooperation with an informal support system – ACT	3	5	5	5	5	5	5	5	5	5	4	5	5	5	5	5	4.81	0.54
55. Cooperation with an informal support system – non-ACT	4	4	2	5	4	5	5	5	5	5	4	5	5	5	5	4	4.50	0.82
Average score Community care	*4.20*	*4.20*	*4.00*	*4.60*	*4.80*	*5.00*	*4.80*	*5.00*	*5.00*	*4.60*	*4.00*	*4.40*	*5.00*	*5.00*	*4.73*	*4.60*	*4.62*	*0.36*
**Monitoring**																		
56. Routine outcome monitoring (ROM) – instruments	3	2	4	3	4	4	3	2	4	4	4	2	4	1	4	3	3.19	0.98
57. Use of ROM	3	3	2	2	3	2	2	2	2	2	3	2	3	2	3	3	2.44	0.51
58. FACT quality improvement	5	4	4	3	5	3	5	3	2	4	4	2	4	4	1	2	3.44	1.21
Average score Monitoring	*4.20*	*3.00*	*3..33*	*2.67*	*4.00*	*3.00*	*3.33*	*2.33*	*2.67*	*3.33*	*3.67*	*2.00*	*3.67*	*2.33*	*2.67*	*2.67*	*3.05*	*0.63*
**Professional development**																		
59. Reflective practice	5	5	1	5	1	5	1	5	1	5	5	5	5	5	1	5	3.75	1.92
60. Training FACT- and related subjects	5	2	5	5	4	5	5	5	5	5	5	5	5	5	5	5	4.75	0.78
61. Structural focus on developmental- and recovery-oriented practice	4	2	3	4	4	2	3	4	4	3	4	3	4	2	2	2	3.12	0.89
62. Team spirit	5	4	3	4	4	3	4	4	4	4	4	3	5	4	4	4	3.94	0.57
Average score Professional development	*4.75*	*3.25*	*3.00*	*4.50*	*3.25*	*3.75*	*3.25*	*4.50*	*3.50*	*4.25*	*4.50*	*4.00*	*4.75*	*4.00*	*3.00*	*4.00*	*3.89*	*0.62*
**Total score Youth FACTS**	4.53	3.89	3.73	4.24	4.06	4.10	4.24	4.11	4.27	4.23	4.47	3.87	4.47	4.18	4.29	4.03	4.17	0.22

Note. [1]Restorative Time-Out (in Dutch: 'bed op recept') is a practice in Dutch psychiatric care where clients who sense an impending crisis are offered the option to spend a calm night in a clinic.
