# Supplementary material for: Examining Youth Flexible ACT Model Implementation in the Netherlands
Source: Community Ment Health J. 2024 Mar 22;60(6):1081–93. doi: 10.1007/s10597-024-01260-z (PMC11199218; doi:10.1007/s10597-024-01260-z)
Supplement: Supplementary file 2 — Supplementary file2 (PDF 229 KB) [file 10597_2024_1260_MOESM2_ESM.pdf]

Online Resource 2

Content of Care Questionnaire

Content of Care Questionnaire (starting from measurement T1)

We would like to know what type of care your client is currently receiving or has received in the past six months.

1. Has the care by the Youth Flexible ACT team been ended?

□ No, proceed to question 3

□ Yes

□ If yes, since when? □□ □□□□ (month/year)

2. What is the reason that your client no longer receives care from the Youth Flexible ACT team?

□ Successfully completed, no further treatment needed

□ Successfully completed, regular care or basic mental health care sufficient

□ Successfully completed, transferred to Adult Flexible ACT care

□ Prematurely closed, Youth Flexible ACT team and the client agreed that Youth Flexible ACT cannot provide adequate or suitable care

□ Prematurely closed, unilaterally by the client

□ Prematurely closed, unilaterally by the Youth Flexible ACT team

□ Juvenile detention centre

□ Moved outside the region

□ Disappeared/inaccessible

□ Other; namely………

3. Does your client currently have a family member receiving Flexible ACT care as well?

□ Yes

  □ Brother or sister

  □ Parent

□ Other; namely………

□ No

4. Has your client been admitted to an inpatient unit, such as in mental health care, forensic care, or addiction care, in the past six months?

□ Yes. This was a planned admission as part of the treatment. Youth Flexible ACT team initiated this.

- □ Number of times:……….
- □ Number of days:……….

□ Yes. This was an unplanned admission (e.g., through crisis services).

- □ Number of times:……….
- □ Number of days:……….

□ No, not admitted

□ Unknown

5. What type of care has your client received from the Youth Flexible ACT team in the past six months?

□ Diagnostic assessment

- □ Data file review
- □ Social history assessments
- □ Developmental assessments
- □ Psychodiagnostic assessment
    - □ Intelligence testing
    - □ Personality assessment
    - □ Neuropsychological assessment
    - □ Other, namely:……….
- □ School observation
- □ Psychiatric assessment
- □ Other, namely:…………

□ Treatment intervention

- □ Psychoeducation
- □ Practical, supportive contacts
    - □ Finances
    - □ Housing
    - □ School/work

□ Daytime activities

□ Other, namely:……….

□ Individual treatment intervention

□ Cognitive Behavioral Therapy

□ Eye Movement Desensitization and Reprocessing

□ Schema therapy

□ Emotion Regulation Therapy

□ Aggression Regulation Therapy

□ Psychomotor Therapy

□ Substance abuse treatment

□ Expressive therapy

□ Other, namely:……….

□ Group treatment intervention

□ Social skills training

□ Emotion regulation training

□ Aggression regulation training

□ Emotion Regulation Therapy

□ Resilience training

□ Other, namely:……….

□ Family interventions

□ Parental counseling

□ Family systemic therapy

□ Functional Family Therapy

□ Multi-Systemic Therapy

□ Other, namely:……….

□ Consults with a peer support worker

□ Pharmacological treatment (psychiatrist)

6. Was the care for your client scaled-up / intensified for a period within the past six months?

□ No

□ Yes, due to:

□ Increase in symptoms, systemic issues, disturbance, or neglect

□ Care avoidant behavior

□ Psychiatric inpatient admission

□ Change of treatment (e.g., different medication or therapy)

□ Life events

7. What is the highest frequent level of care that your client has received from the Youth Flexible ACT team in the past six months?

□ Less than one appointment every 14 days

□ One appointment every 14 days

□ One appointment every 7 days

□ Multiple appointments in the week

□ Daily appointments (5 working days)
