# Supplementary figures and images for: Examining Youth Flexible ACT Model Implementation in the Netherlands
Source: Community Ment Health J. 2024 Mar 22;60(6):1081–93. doi: 10.1007/s10597-024-01260-z (PMC11199218; doi:10.1007/s10597-024-01260-z)

### Online Resource 3 *Flow Diagram of Study Participants*

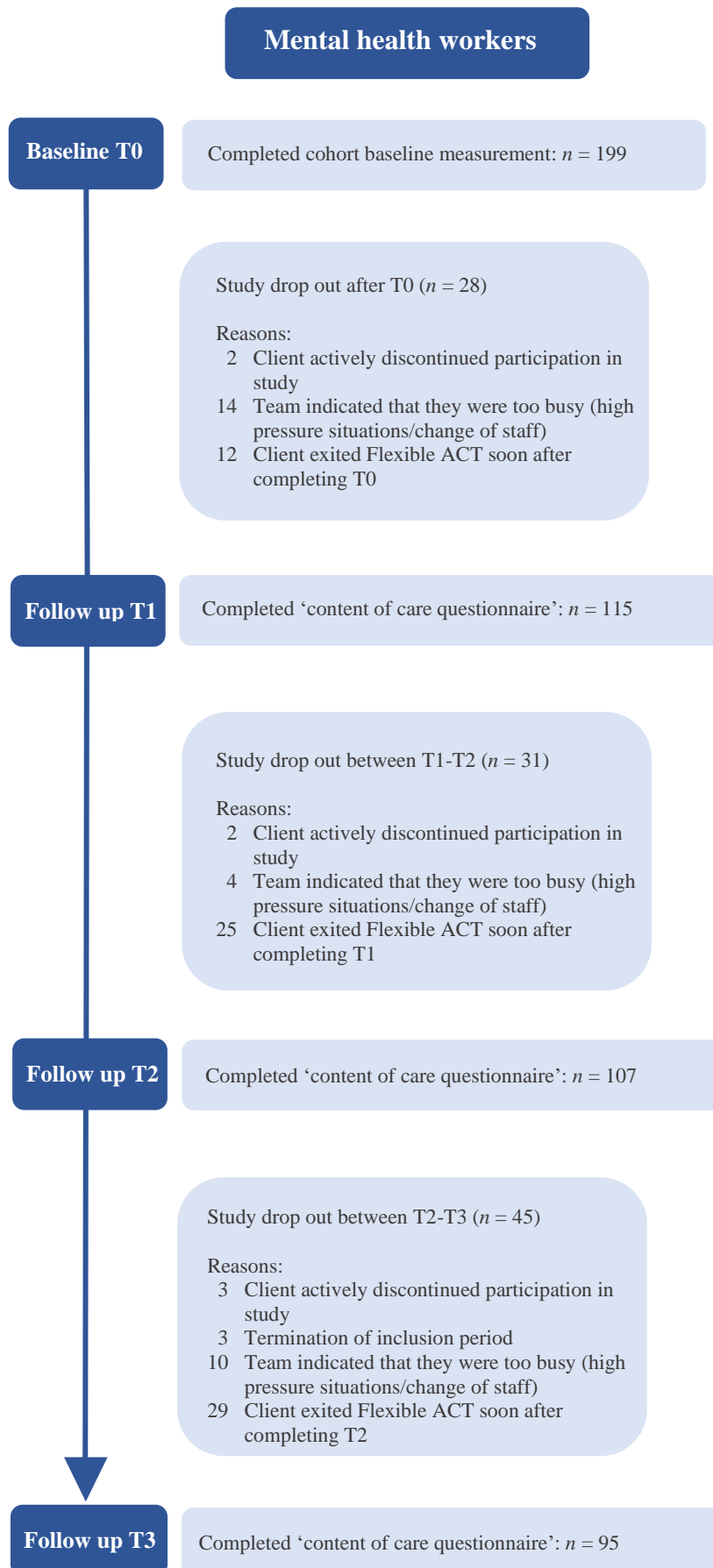

Supplement: Supplementary file 3 — Supplementary file3 (PDF 84 KB) [file 10597_2024_1260_MOESM3_ESM.pdf]
